# Presenilin-1 Affects Melanoma Cell Behavior in an Amyloid Precursor Protein-Rich, Alzheimer’s Disease-like, Microenvironment

**DOI:** 10.3390/ijms27135885

**Published:** 2026-06-30

**Authors:** Gustavo Untiveros, Rebecca St. Germain, Briana Barrington, Stacy Ann Kujawa, Alesia V. Prakapenka, Luigi Strizzi

**Affiliations:** 1 Department of Pathology, College of Graduate Studies, Midwestern University, 555 31st Street, Downers Grove, IL 60515, USA; guntiv@midwestern.edu (G.U.); rebecca.stgermain06@gmail.com (R.S.G.); briana.barrington@midwestern.edu (B.B.); 2 Biomedical Sciences Program, College of Graduate Studies, Midwestern University, 555 31st Street, Downers Grove, IL 60515, USA; skujaw@midwestern.edu (S.A.K.); apraka@midwestern.edu (A.V.P.)

**Keywords:** melanoma, Alzheimer’s, amyloid, brain, cancer growth

## Abstract

Recent studies report inverse relationships between the incidence of Alzheimer’s Disease (AD) and certain malignancies, including melanoma. This has encouraged research into factor(s) in AD that can exert antitumor effects. Presenilin-1 (PS-1) is part of the enzymatic complex that cleaves amyloid precursor protein (APP) into amyloid-beta (Aβ) products that are linked to the neuronal damage seen in AD. PS-1 can also degrade β-catenin and reduce the effectiveness of the “wingless-related integration” (WNT) signaling pathway. Little is known about the relationship between the AD microenvironment, PS-1, and melanoma. We hypothesize that melanoma growth in AD depends on the degree of PS-1-dependent processing of APP into cytotoxic Aβ by melanoma cells. To determine how melanoma reacts to an APP-rich, AD-like microenvironment, PS-1-high (WM1552C) and PS-1-low (C8161) melanoma cells were treated with soluble recombinant human APP (rhAPP). We found that rhAPP treatment significantly reduced cellular activity in WM1552C but not in C8161 cells. Moreover, Aβ products were significantly higher in conditioned media from rhAPP-treated WM1552C compared to controls. Treatment with PS-1 inducing DAPT or PS-1 function inhibiting MRK-560 reversed the effects of rhAPP treatment, respectively in C8161 and WM1552C cells. Furthermore, we found that migration of WM1552C was significantly reduced in the presence of either soluble rhAPP or mouse AD brain tissue, compared to C8161, suggesting that in WM1552C the combination of PS-1 activity and the presence of APP/Aβ in the microenvironment interferes with cell migration. In summary, PS-1 function may predict how melanoma will grow in an APP/Aβ-rich microenvironment, such as AD.

## 1. Introduction

Alzheimer’s Disease (AD) is the most common form of dementia affecting adults and is one of the leading causes of death in the US [1]. Interestingly, patients diagnosed with AD appear to have a lower risk for developing certain cancers [2]. In fact, studies have described an inverse relationship between AD and skin cancers, including melanoma [3].

Amyloid Precursor Protein (APP) is a transmembrane protein that plays a physiologic role in neuronal signaling, synaptic function and plasticity [4,5]. In AD, APP accumulates in the brain [6] and is processed by the gamma-secretase (GS)/Presenilin-1 (PS-1) complex [7] into cytotoxic amyloid beta (Aβ) proteins that can induce neuronal damage and inflammation observed in AD [8]. The cytotoxic effects caused by these Aβ products range from interference with mitosis and reduction in cell cycle to increased production of free radical oxygen species, which can damage mitochondria, leading to apoptosis [9,10]. Although Aβ40 production in AD is much greater than Aβ42, the predominant isoform in the brain linked to AD is the more cytotoxic Aβ42. Variants of APP can be found in other peripheral tissues, where they participate in diverse cellular functions [11,12,13,14]. Furthermore, normal processing of peripheral APP may also be compromised in AD. For instance, activated platelets from AD patients have been shown to produce higher levels of peripheral Aβ products [15], suggesting that the APP-rich, amyloidogenic microenvironment is a systemic alteration in AD with potential effects outside the central nervous system.

Previous studies have shown that mutations in PS-1 can disrupt normal APP cleavage and clearance of Aβ degradation products [16]. This alteration can also interfere with multiple cellular signaling pathways, including those that regulate GS activity, thereby exacerbating the dysregulated APP processing [17]. PS-1 also plays an important role in regulating ‘wingless-related integration’ (WNT) signaling via the GS-independent inactivation of β-catenin [18]. The WNT signaling pathway is known to regulate important cellular processes involved in both normal development and tumorigenesis [19,20]. These events include cell adhesion and polarity, proliferation, migration, and invasion [21,22], which have also been shown to play a role in melanoma [23]. Interestingly, PS-1 was shown to be poorly expressed in WNT-active, aggressive melanoma cells compared to non-aggressive melanoma cells that exhibited relatively higher PS-1 expression [24], suggesting that the degree of PS-1 expression and effects on WNT signaling may influence melanoma aggressiveness.

Since PS-1 dysfunction can contribute to the accumulation of cytotoxic Aβ products in the APP-rich microenvironment of the AD brain, aberrant PS-1 activity could affect melanoma growth in AD patients. A study found that human melanoma cells extracted from non-AD patient brain metastases express PS-1 and are thus capable of cleaving melanoma-derived APP into Aβ isoforms [25]. In that study, it was concluded that melanoma cells in these brain metastases create an anti-inflammatory environment via the production of Aβ, allowing the melanoma cells to proliferate despite the local host anti-tumor immune response. However, there is no evidence to date regarding whether Aβ isoforms induce cytotoxic effects on the melanoma cells, themselves.

The goal of this study is to clarify the dynamic relationships between APP processing, Aβ production, and potential cytotoxicity in melanoma cells, and how this relationship may influence melanoma survival in an APP-rich environment, such as AD. Results from this study may further our understanding of the inverse relationship between AD and cancers, lead to the discovery of novel drivers of melanoma proliferation, and provide insight into strategies for targeting these drivers with innovative anti-melanoma therapies.

## 2. Results

### 2.1. Melanoma Cells Express Variable Amounts of Endogenous APP

**Figure 1:**
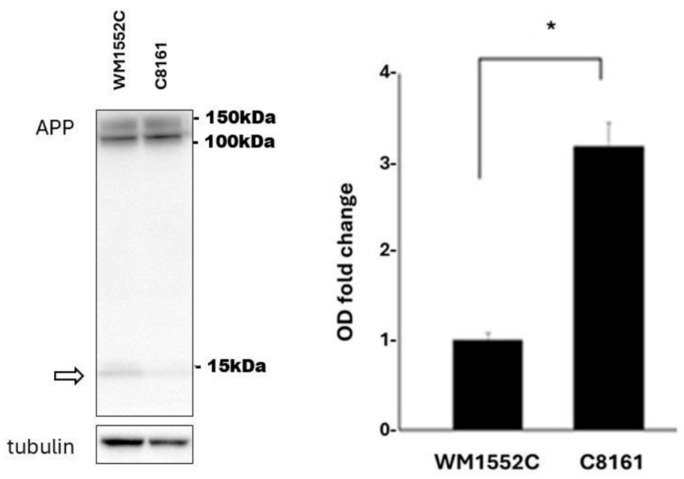
Endogenous APP expression in melanoma cells. Representative WB showing APP expression in WM1552C and C8161 melanoma cells. A higher expression of a lower-molecular-weight protein was occasionally detected in WM1552C compared to C8161 (arrow). The histogram summarizing the densitometric analysis of the optical density (OD) of the WB bands shows that PS-1-high WM1552C melanoma cells express a significantly lower amount of APP compared to PS-1-low C8161 melanoma cells. OD data are presented as the mean fold change in WM1552C ± SEM, of 3 independent experiments. (unpaired *t*-test, * *p* = 0.001).

A previous study reported that melanoma cells obtained from non-AD brain metastasis express endogenous APP [25]. To determine whether the melanoma cells used in our study also expressed endogenous APP, we performed Western blot (WB) analysis on cell lysates obtained from WM1552C and C8161 cells. The WB results showed significantly higher expression of endogenous APP in PS-1-low C8161 cells compared to PS-1-high WM1552C cells (3.17 ± 0.270 fold of WM1552C, unpaired *t*-test, *p* = 0.001) (Figure 1). WB results also detected higher expression of low-molecular-weight protein, comparable to Aβ, in WM1552C compared to C8161 cells (Figure 1). These findings suggest that the difference in endogenous APP expression may result from the increased consumption of this APP as it is degraded in Aβ products due to the relatively higher expression of PS-1 in WM1552C compared to C8161 cells.

### 2.2. Melanoma Cells Produce APP Degradation Products, Aβ40 and Aβ42

**Figure 2:**
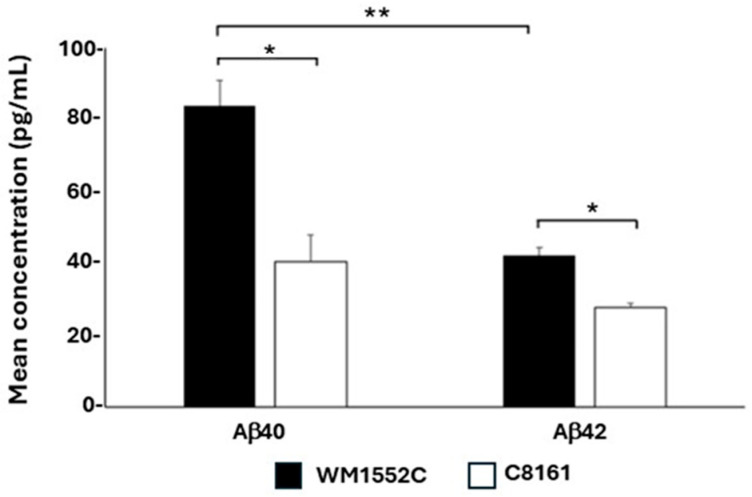
Aβ40 and Aβ42 levels in conditioned media from melanoma cells. ELISA results show that conditioned media from WM1552C contained significantly higher levels of Aβ40 (unpaired *t*-test, *p* = 0.006) and Aβ42 (unpaired *t*-test, * *p* = 0.001) compared to conditioned media from C8161. Aβ40 levels are also significantly higher than Aβ42 in the conditioned media of WM1552C (** paired *t*-test, *p* = 0.01). Data is represented as mean concentrations (pg/mL) ± SEM, of at least 3 independent experiments.

PS-1 plays a role in the processing of APP into the main cleavage products, Aβ40 and Aβ42 [8]. To investigate whether different PS-1 expression levels affect Aβ40 and Aβ42 production in melanoma cells, we performed ELISA to detect and quantify these APP cleavage products in the conditioned media collected from WM1552C and C8161 melanoma cells. ELISA results showed detection of both Aβ40 and Aβ42 in the conditioned media from both WM1552C and C8161 cells (Figure 2). However, the level of Aβ40 was significantly higher in the conditioned media from WM1552C compared to C8161 (WM1552C = 83.8 ± 7.1 pg/mL versus C8161 = 40.4 ± 7.4 pg/mL, unpaired *t*-test, *p* = 0.006) (Figure 2). Similarly, ELISA results showed that Aβ42 was also significantly higher in the conditioned media from WM1552C compared to C8161 (WM1552C = 42.2 ± 2.2 pg/mL versus C8161 = 27.6 ± 1.3 pg/mL, unpaired *t*-test, *p* = 0.001). Also, in conditioned media from WM1552C cells, Aβ40 levels were significantly higher than Aβ42 levels (paired *t*-test, *p* = 0.01) (Figure 2). These results suggest that, compared to PS-1-low C8161 melanoma cells, PS-1-high WM1552C melanoma cells exhibit greater depletion of cytosolic APP due to its increased processing into extracellular Aβ40 and Aβ42.

### 2.3. Effect of Exogenous rhAPP on PS-1 Expression in Melanoma Cells

**Figure 3:**
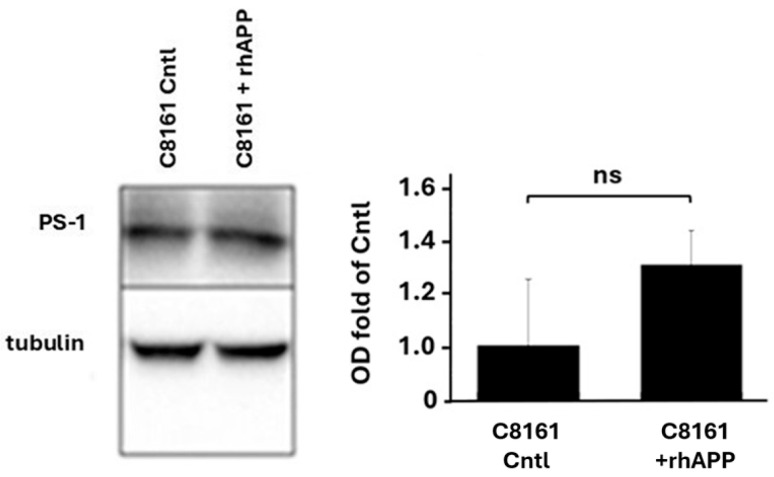
PS-1 expression in C8161 cells treated with rhAPP. Representative WB and corresponding histogram of densitometric analysis of WB bands show no significant change in PS-1 expression in C8161 cells after 48 h treatment with 100 nM of rhAPP (C8161 + rhAPP) compared to untreated control (Cntl) (C8161 Cntl). Data are presented as the mean fold change in OD relative to control ± SEM, of 3 independent experiments. (ns = non-significant, paired *t*-test).

Substrate induction is a well-known process whereby the cellular expression of a certain enzyme can increase upon exposure to the corresponding substrate [26]. Our results show no significant change in PS-1 expression in C8161 melanoma cells treated for 48 h with 100 nM of exogenous rhAPP compared to untreated control (Figure 3) indicating that exposure to exogenous rhAPP does not increase PS-1 expression in the melanoma cells tested.

### 2.4. Processing of APP into Aβ40 and Aβ42 Is Dependent on PS-1 Expression in Melanoma Cells

**Figure 4:**
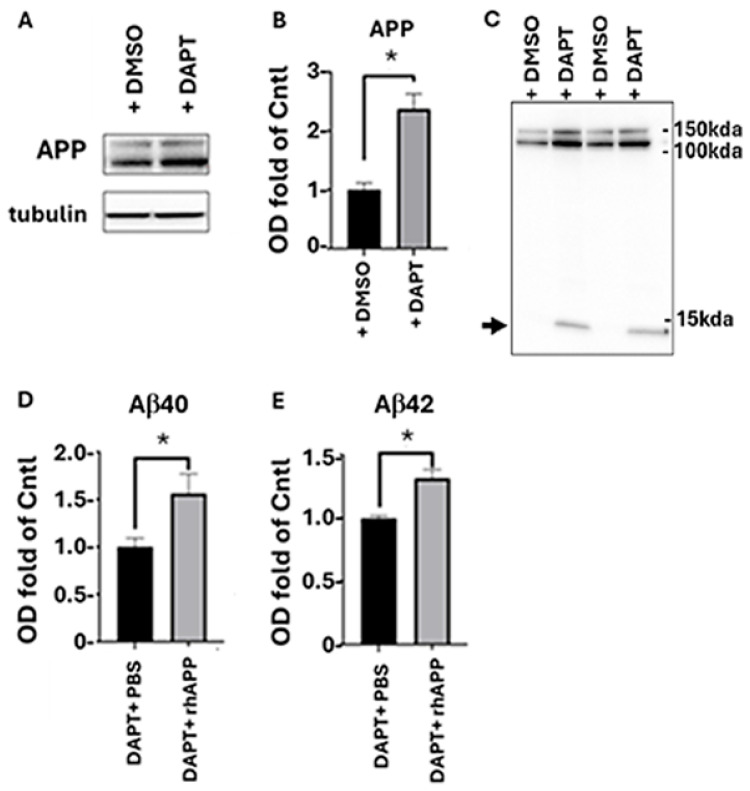
Effect of DAPT treatment on APP and Aβ expression in C8161 cells. Densiometric analysis of WB bands in (**A**) shows a significant increase in APP expression in C8161 melanoma cells after 48 h treatment with 30 μM DAPT compared to DMSO control (paired *t*-test, * *p* = 0.02) (**B**). Representative WB in (**C**) from two independent experiments also shows concomitant expression of low-molecular-weight protein in DAPT-treated C8161, presumably APP degradation product of less than 20 kDa (arrow), in DAPT-treated C8161 cells that is not detected in control cells. C8161 cells treated with 30 μM DAPT ± 100 nM rhAPP show significant increased production of Aβ40 (paired *t*-test, * *p* = 0.04) (**D**) and Aβ42 (paired *t*-test, * *p* = 0.04) (**E**) in conditioned media from DAPT + rhAPP-treated cells compared to DAPT + PBS-treated controls. Data are presented as the mean fold OD of control ± SEM of at least 3 independent experiments.

To demonstrate that APP processing into Aβ40 and Aβ42 is dependent on PS-1 expression in melanoma cells, we treated PS-1-low C8161 cells with DAPT, which has previously been shown to increase PS-1 expression in C8161 cells [24]. This allowed us to assess whether increased PS-1 expression enhances the ability of C8161 cells to process APP into Aβ products. WB results of lysates from C8161 treated for 48 h with 30 μM of DAPT (Figure 4A) showed an unexpected significant increase in endogenous APP expression compared to DMSO control cells (2.4 ± 0.26 fold of control, paired *t*-test, *p* = 0.02) (Figure 4B). Furthermore, WB results show protein with a molecular weight below 20 KDa, consistent with suspected APP degradation products in the DAPT-treated C8161 cells that were not detected in control cells (Figure 4C). In fact, ELISA results of conditioned media collected from DAPT + rhAPP-treated C8161 compared to DAPT + PBS-treated control cells demonstrated a significant 1.56 fold increase in Aβ40 (DAPT + PBS control = 16.7 ± 1.5 pg/mL versus DAPT + rhAPP = 26.1 ± 3.5 pg/mL, paired *t*-test, *p* = 0.04) and a significant 1.36-fold increase in Aβ42 (DAPT + PBS control = 103.5 ± 2.2 pg/mL versus DAPT + rhAPP = 141.1 ± 9.5 pg/mL, paired *t*-test, *p* = 0.04). These data demonstrate that DAPT-induced PS-1 expression in melanoma cells leads to a compensatory increase in intracellular APP, as it is being processed via PS-1-driven cleavage into Aβ40 and Aβ42.

### 2.5. Reduced Activity of Melanoma Cells in APP Correlates with PS-1 Dependent Production of Aβ40 and Aβ42

**Figure 5:**
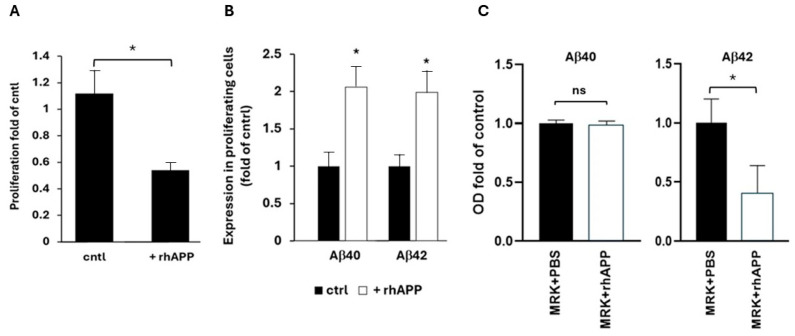
Effect of exogenous rhAPP on WM1552C melanoma cells. (**A**) MTT assay results indicate significantly decreased cellular activity in WM1552C melanoma cells following treatment with 100 nM of rhAPP (+rhAPP) compared to untreated control cells (cntl) (paired *t*-test, * *p* = 0.02). (**B**) ELISA results show that WM1552C cells produced significantly higher levels of Aβ40 (paired *t*-test, * *p* = 0.003) and Aβ42 (paired *t*-test, * *p* = 0.03) compared to untreated cntl cells. (**C**) ELISA results show that, compared to control (MRK + PBS), WM1552C cells co-treated with 100 nM MRK-560 (MRK) and 100 nM of rhAPP no longer show increased production of Aβ40 (ns = non-significant, paired *t*-test), but show a significant reduction in Aβ42 production (paired *t*-test, * *p* = 0.006). Data are presented as the mean fold OD of control ± SEM of at least 3 independent experiments.

Although APP is not cytotoxic per se, the accumulation of its cleavage products, Aβ40 and Aβ42, can cause a wide range of adverse cellular effects, from reduced cell metabolism and prolonged cell cycle to cell death [9,10]. Thus, cell proliferation in an APP-rich microenvironment may ultimately depend on the extent to which cells produce Aβ40 and/or Aβ42 via degradation of APP. To determine the differences in PS-1 expression between WM1552C and C8161 and, consequently their ability to generate Aβ40 and Aβ42 that can affect cellular activity, we treated melanoma cells with exogenous rhAPP and performed an MTT assay. Although we did not detect any significant difference in results between rhAPP-treated and untreated C8161 cells the rhAPP-treated WM1552C cells showed a significant reduction in cellular activity compared to untreated control cells (0.48 ± 0.06 fold of control, paired *t*-test, *p* = 0.02) (Figure 5A). This suggests that WM1552C cells utilize PS-1 to generate Aβ40 and Aβ42, which in turn negatively affect cellular activity.

To confirm that the reduced cellular activity in rhAPP-treated WM1552C cells was due to PS-1-dependent increased processing of rhAPP into cytotoxic Aβ products, we performed ELISA on conditioned media collected from these cells. In the metabolically active fraction identified by the MTT assay, Aβ40 and Aβ42 levels were approximately two-fold greater in rhAPP-treated WM1552C cells compared to untreated controls (Aβ40 control = 71.8 ± 13.6 pg/mL versus Aβ40 + rhAPP = 147.7 ± 19.7 pg/mL, paired *t*-test, *p* = 0.003; Aβ42 control = 22.9 ± 3.3 pg/mL versus Aβ42 + rhAPP = 45.6 ± 6.6 pg/mL, paired *t*-test, *p* = 0.03) (Figure 5B). Thus, WM1552C cells exhibit signs of cellular distress, as evidenced by significantly reduced metabolic activity and increased Aβ40 and Aβ42 production following 48 h exposure to rhAPP.

Treatment of the rhAPP-exposed WM1552C cells with the PS-1 function inhibitor MRK-560 abolished the ability of these cells to generate Aβ40 from exogenous rhAPP (Figure 5C). Moreover, MRK-560 treatment significantly reduced the ability of WM1552C, by more than half, to produce Aβ42 from rhAPP (MRK-560 + PBS control = 10.5 ± 1.2 pg/mL versus MRK-560 + rhAPP = 4.3 ± 1.7 pg/mL, paired *t*-test, *p* = 0.006) (Figure 5C). In addition, WM1552C cells treated with MRK-560 no longer exhibited the previous negative effect on cellular activity following rhAPP exposure. Overall, these results suggest that PS-1-expressing melanoma cells show a decreased cellular activity in the presence of exogenous APP due to increased PS-1-dependant processing of APP into cytotoxic Aβ products.

### 2.6. PS-1 Expression Is Associated with Reduced Migration Towards APP/Aβ Rich Microenvironments

**Figure 6:**
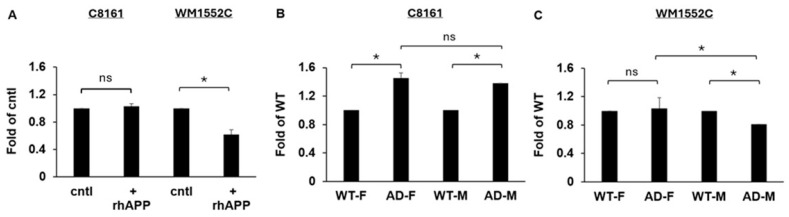

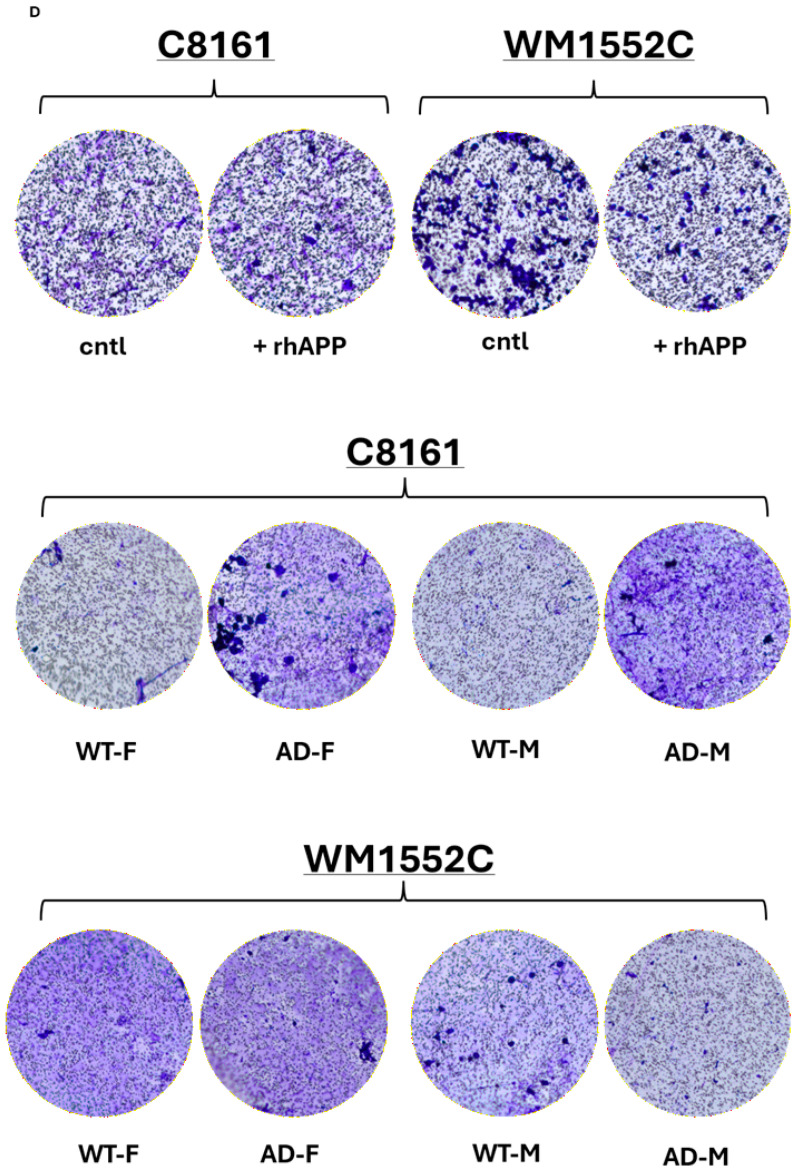
Migration of melanoma cells in an APP-rich microenvironment. (**A**) Treatment with 100 nM of exogenous soluble rhAPP did not affect the migration of C8161 cells compared to untreated control, but significantly reduced migration of WM1552C compared to untreated control (paired *t*-test, * *p* = 0.02). (**B**) Migration of C8161 cells was significantly increased towards brain tissue of AD female (AD-F) (paired *t*-test, * *p* = 0.007) and AD male (AD-M) (paired *t*-test, * *p* = 0.03) mice compared to brain tissue from age- and sex-matched wildtype (WT) control mice. No significant (ns) difference was observed between C8161 migration towards AD-F and AD-M brain tissue. (**C**) No significant difference was observed in the migration of WM1552C cells towards AD-F compared to WT-F brain tissue. However, migration of WM1552C cells was significantly reduced towards AD-M compared to WT-M brain tissue (paired *t*-test, * *p* = 0.005). Additionally, migration of WM1552C toward AD-M brain tissue was significantly lower than toward AD-F brain tissue (paired *t*-test, * *p* = 0.03). (**D**) Microphotographs of inserts with stained cells representative of the migration results towards the different types of mice brain tissues used are shown. Data are presented as the mean fold OD of control ± SEM of 4 independent experiments.

To determine whether differences in PS-1 expression affect the ability of melanoma cells to migrate towards an APP-rich microenvironment, a chemotaxis/cell migration assay was performed using WM1552C and C8161 in the presence or absence of rhAPP. Results revealed that while exogenous rhAPP had no significant effect on the migration of C8161 cells compared to control, the presence of rhAPP significantly reduced the migration of WM1552C cells (0.45 ± 0.06 fold of control, paired *t*-test, *p* = 0.02) (Figure 6A).

The chemotaxis/cell migration assay was repeated using brain tissue from sex- and age-matched wildtype (WT) control mice or from matching APP/Aβ-rich brain tissue from AD mice. Results showed a significant increase in C8161 cell migration towards AD-F brain tissue (1.45 ± 0.07 fold of control, paired *t*-test, *p* = 0.007) and AD-M brain tissue (1.38 ± 0.10 fold of control, paired *t*-test, *p* = 0.03) compared to cell migration towards the respective sex-matched WT controls (Figure 6B). No significant difference was observed between C8161 migration towards AD-F and AD-M brain tissues (Figure 6B). For WM1552C cells, no significant change was observed in migration towards AD-F brain tissue compared to WT-F brain tissue (Figure 6C). However, there was a significant decrease in the migration of WM1552C cells towards AD-M brain tissue compared to WT-M brain tissue (0.81 ± 0.02 fold of control, paired *t*-test, *p* = 0.005) (Figure 6C). Notably, the migration of WM1552C cells towards AD-M brain tissue was also significantly lower than migration towards AD-F brain tissue (paired *t*-test, *p* = 0.03) (Figure 6C). These results show that compared to PS-1-low C8161 cells, PS-1-high WM1552C cells exhibit a reduced capacity to migrate towards an APP/Aβ-rich microenvironment, including AD brain tissue.

## 3. Discussion

Recently, studies have shown an inverse relationship between the incidence of AD and certain cancers including melanoma, the most aggressive skin cancer [2,3,27,28]. In this study, we hypothesize that the inverse relationship between melanoma and AD depends on PS-1 expression. We found that endogenous APP expression was higher in PS-1-low, aggressive C8161 melanoma cells compared to PS-1-high, less-aggressive, WM1552C melanoma cells. These results suggested that the increased expression of PS-1 in WM1552C cells enhances the processing of endogenous APP into Aβ products. Consistent with this, ELISA results revealed that conditioned media from WM1552C cells contained significantly higher levels of cytotoxic Aβ40 and Aβ42 compared to C8161 cells. Furthermore, cell migration assays indicated that PS-1 expression negatively impacted the migratory behavior of WM1552C towards rhAPP and mice AD brain tissue.

Although progress has been made in melanoma therapy through targeted signaling pathways and immunotherapy, patient survival remains poor. Disease progression with metastatic spread, despite therapy, is the major cause of death in melanoma patients [29]. Thus, further research aimed at preventing melanoma progression and metastasis may improve patient outcomes. Studying how one disease negatively influences another—for example, how certain blood disorders confer protection against parasitic infestations such as malaria [30]—could provide insight into mechanisms by which the pathogenic processes of one condition interfere with another. Therefore, the molecular mechanism(s) underlying the formation of an APP/Aβ-rich microenvironment in AD may influence melanoma growth.

PS-1, the enzymatic partner of GS, has been shown to play a role in the processing of endogenous APP into Aβ40 in melanoma, facilitating tumor growth in brain metastases, suggesting that this mechanism could promote melanoma proliferation in the AD brain [25]. However, epidemiologic data demonstrate an inverse association between some cancers, including melanoma and AD [3,31], indicating that antagonistic molecular mechanisms may exist between AD and melanoma. Beyond its role with GS, PS-1 can also degrade β-catenin and negatively regulate the WNT signaling pathway, which is involved in cancer progression [18]. In support of this, our previous study reported that advance-stage melanoma tissues show lower PS-1 expression compared to early-stage [24]. Furthermore, in that same study, aggressive melanoma cells with active WNT signaling showed lower PS-1 expression compared to poorly aggressive melanoma cells with reduced WNT activity, but higher PS-1 expression. These findings suggest that PS-1 may regulate melanoma progression through WNT signaling and influence how melanoma cells respond to APP-rich environments. However, the relationship between AD, PS-1, and melanoma remains poorly understood.

Since AD is linked to cytotoxic effects of Aβ40 and Aβ42 in the brain, we investigated whether the increased PS-1 expression in WM1552C cells would enhance the production of these cytotoxic proteins and affect cellular growth in an APP-rich microenvironment, like that of the AD brain. We found that cellular proliferation was significantly reduced in WM1552C cells exposed to exogenous APP, suggesting that increased PS-1 expression promotes the production of cytotoxic Aβ40 and Aβ42 that negatively affected cell growth.

This present study is the first to suggest that the ability of melanoma cells to grow in an APP-rich microenvironment, such as the AD brain, depends on PS-1 expression. Our findings indicate that melanoma cells with high PS-1 expression may have a proliferative disadvantage in AD brains due to their enhanced ability to process APP into cytotoxic Aβ40 and Aβ42. Consistent with this, PS-1-high WM1552C cells were less attracted to mice AD brain tissue compared to PS-1-low C8161 cells. Notably, WM1552C migration was significantly reduced toward male AD brain tissue compared to female AD brain tissue.

Previous studies have shown that female AD mice exhibit higher Aβ burden and faster accumulation compared to age-matched males [32]. Additionally, Aβ has been reported to exert a negative feedback effect on PS-1 activity, such that increased Aβ accumulation reduces PS-1 function [33]. Therefore, the higher Aβ burden in female mice AD brain may inhibit PS-1 activity in WM1552C cells, limiting further Aβ production and reducing its cytotoxic effects including negatively affecting cell migration. This may explain why the reduction in migration of WM1552C cells was more pronounced towards male AD brain tissue compared to female AD brain tissue. In contrast, C8161 cells, which have low PS-1 expression, would be less capable of processing APP into Aβ and thus less affected by its cytotoxic effects, while also exhibiting increased aggressiveness due to enhanced WNT signaling [24].

The findings reported here may help explain some of the epidemiological observations of an inverse relationship between AD and melanoma by highlighting the role of PS-1 in modulating melanoma responses to APP. Our data demonstrates that PS-1 expressing melanoma cells are more likely to process environmental APP into cytotoxic Aβ products, thereby reducing their proliferative capacity in APP-rich environments, such as the AD brain. In contrast, melanoma cells with low or absent PS-1 expression may be less affected by exogenous APP and therefore retain a greater capacity to proliferate in an APP-rich microenvironment.

A limitation of this study is that it does not account for the progressive nature of AD or the dynamic changes in the AD brain microenvironment over time, including inflammation and immune responses, which may influence melanoma growth. Nevertheless, from a therapeutic perspective, strategies aimed at increasing PS-1 expression in melanoma cells may reduce tumor progression by both inhibiting WNT signaling, as previously demonstrated [24], and limiting metastatic spread to APP-rich tissues. In fact, in addition to the brain, where APP plays a physiologic role for neuronal synaptic function and plasticity, variants of APP can be found in other peripheral tissues including liver, pancreas, adipose tissue and muscle where it can participate in cell signaling and adhesion, immune function, tissue repair and metabolic processes [12,13,14]. Therefore, PS-1 expression may also influence melanoma metastasis to these tissues. Moreover, because this data shows that the ability for melanoma to proliferate in AD appears to depend on PS-1 expression, future epidemiological studies examining the incidence of melanoma and AD would require additional stratification of melanoma cases based on PS-1 expression. Also, if melanoma does arise in patients with AD, and given that AD appears to be a systemic condition, it would be of interest to note all organs involved in melanoma spread to see whether PS-1 expression and function in melanoma cells is associated with a predilection towards specific metastatic sites.

## 4. Methods and Materials

### 4.1. Cell Culture

The human melanoma cell lines WM1552C (Rockland Immunochemicals, Limerick, PA, USA) and C8161 (CVCL_6813) (a kind gift from Dr. Richard Seftor, University of West Virginia, USA) have been previously characterized according to their PS-1 expression [24]. WM1552C was used as the PS-1-high, non-aggressive melanoma cell line and C8161 as the PS-1-low, aggressive melanoma cell lines. Both cell lines were maintained in RPMI1640 medium (25-506, GenClone, San Diego, CA, USA) supplemented with 5% FBS (97068-085, Seradigm, Batavia, IL, USA) and grown in an incubator set at a 5% CO_2_ atmosphere and 37 °C.

### 4.2. Protein Extraction and Western Blot Analysis

C8161 cells, prior to cell lysis, were grown at 100,000 cells/well at 2 mL/well in 6-well plates and treated for 48 h in RPMI1640 containing 5%FBS to the indicated final concentrations: media only for baseline; 100 nM rhAPP prepared as a 1:100 dilution in PBS from a 0.1 mg/mL stock (842601, Biolegend, San Diego, CA, USA) with a matching PBS vehicle control; or 30 μM N-[N-(3,5-difluorophenacetyl)-L-alanyl]-S-phenylglycine t-butyl ester prepared from a 10 mM stock (DAPT, A8200, ApexBio, Houston, TX, USA), previously shown to increase PS-1 expression in these cells [24], with a matching DMSO vehicle control. Similarly, WM1552C cells were grown at 100,000 cells/well at 2 mL/well in 6-well plates for 48 h to serve for baseline assessment.

Lysates were then collected using RIPA buffer (PI8990, Pierce, Waltham, MA, USA) containing protease inhibitors (PIA32959, Pierce, Waltham, MA, USA). The resulting lysates were quantified using standard BCA quantification (23227, Pierce, Waltham, MA, USA). SDS-Page electrophoresis was used to separate 30 μg of protein per sample. Proteins were then transferred to PVDF membranes (IPVH00010, Millipore, Burlington, MA, USA). After protein transfer, membranes were washed with tris-buffered saline with 0.1% Tween 20 (TBST) and then blocked with 5% nonfat dry milk (NFDM) or 5% bovine serum albumin (BSA) for 1 h at room temperature. Membranes were then incubated overnight at 4 °C with adequate dilutions of primary antibodies in their respective blocking buffer. The listed primary antibodies and dilutions were used: goat anti-presenilin (AF166 R&D Systems, Minneapolis, MN, USA)/2.6:1000, rabbit anti-α-tubulin (2144S, Cell Signaling, Danvers, MA, USA)/1:2000, rabbit anti-APP (76600, Cell Signaling, Danvers, MA, USA)/1:2000. Membranes were then washed with TBST and incubated for 1 h with the appropriate conjugated secondary antibodies: anti-mouse (NA931, GE Amersham, Marlborough, MA, USA)/1:5000, anti-rabbit (NA934, GE Amersham)/1:5000, and anti-goat (HADF109, R&D Systems, Minneapolis, MN, USA)/1:5000. After washing with TBST, membranes were incubated in ECL (RPN2235, Cytiva, Wilmington, DE, USA) for 5 min and images of bands captured using Universal Chemidoc II (Bio-Rad, Hercules, CA, USA).

### 4.3. MTT Assay, ELISA, and Cell Treatment Experiments

To determine whether the growth of melanoma cells in an APP enriched microenvironment depended on the degree of PS-1 expression, 20,000 PS-1-high WM1552C or PS-1-low C8161 cells were seeded in a 48-well plate containing 0.2 mL of culture media supplemented with 100 nM rhAPP (842601, Biolegend, San Diego, CA, USA) or PBS, used as control. After 48 h incubation, the effects on cell proliferation were determined by using a standard 4,5-dimethylthiazol-2yl-2,5-diphenyltetrazolium bromide (MTT) assay (PR-G4000, Promega, Madison, WI, USA). We used MTT because it assesses mitochondrial activity in viable cells and is thus capable of reflecting in vitro cellular cytotoxicity [34]. Briefly, 30 μL/well of MTT dye solution was added and allowed to incubate for 4 h at 37 °C after which, we added 200 μL/well of Stop/Mix solution and allowed the plate to incubate for 1 h at 37 °C. The intensity of the MTT staining result was read at 570 nM using the Perkin’s Elmer 2300 Enspire plate reader.

Enzyme-Linked Immunosorbent Assay (ELISA) was used to determine baseline production of Aβ40 and Aβ42 (DAB140B, DAB142, R&D Systems, Minneapolis, MN, USA) in conditioned media collected from WM1552C and C8161 cells grown at 100,000 cells/well at 2 mL/well in 6-well plates for 48 h. ELISA was also performed on conditioned media of C8161 cells treated for 48 h with 30 μM DAPT (A8200, ApexBio, Houston, TX, USA) and a PBS vehicle control or co-treated with 30 μM DAPT and 100 nM rhAPP to determine whether there were changes in the production of Aβ40 and Aβ42. Similarly, we collected conditioned media for ELISA from WM1552C cells grown as above and treated with 100 nM rhAPP or matching PBS control. Finally, conditioned media was also collected from WM1552C cells cotreated with either 100 nM of the PS-1 function inhibitor MRK-560 [35] prepared as a 1:100 dilution in DMSO from a 3.86 mM stock (SML3330, Millipore Sigma, St Louis, MO, USA) along with a PBS control match for 100 nM rhAPP or cotreated with both 100 nM rhAPP and 100 nM MRK-560.

### 4.4. Melanoma Cell Migration Assay

To determine whether PS-1 expression can affect the ability of melanoma cells to migrate in an APP-rich microenvironment, approximately 40,000 cells of C8161 or WM1552C, previously serum starved for 24 h, were seeded in a 24-well cell migration trans well system containing inserts with 3 µm porous membranes (141004, Nunc, Rochester, NY, USA) that allow cells to migrate through. These inserts in turn were placed at a 6.3 mm height in wells containing matching RPMI1640 containing 5%FBS 1.5 mL of media with 100 nM of rhAPP or PBS vehicle control and were allowed to migrate overnight for approximately 16 h.

The same migration assay was repeated with mice brain tissue instead of rhAPP to see how the melanoma cells migrate in the presence of these tissues obtained from 6-month old sex-matched wildtype (WT) C57BL/6J mice (n = 6 female WT mice and n = 6 male WT mice) or from 6-month old sex matched 3xTg-AD mice (n = 6 female AD mice and n = 6 male AD mice). Briefly, mice were deeply anesthetized with intraperitoneal injection of ketamine/xylazine cocktail (120 mg/kg ketamine, 10 mg/kg xylazine) followed by rapid decapitation. After surgical resection of the scalp and exposure of the brain, the brain was extracted from the skull. Immediately following removal, brain was sectioned coronally in a 1 mm brain matrix on ice. Sections were rapidly frozen in liquid nitrogen and stored at −80 °C until further processing. The 3xTg-AD is a transgenic mouse model used to study human AD progression and contains pathogenic levels of the main components linked to human AD, such as APP and Aβ [36]. The number of animals used was determined using a sample size calculator (https://wnarifin.github.io/ssc_web.html) (accessed on 1 July 2024) and based on comparisons made between two groups (female WT versus female AD or male WT versus male AD) using at least 2 technical repeats. All mice were bred in-house, with breeders purchased from Jackson Laboratories (Bar Harbor, ME, USA). Mice were housed with sex-matched littermates on a 12:12 light-dark cycle, with food and water *ad libitum*. All procedures were approved by Midwestern University’s Institutional Animal Care and Use Committee (IACUC) (protocol #05011, approved 24 July 2024).

After approximately 16 h overnight incubation at 5% CO_2_ and 37 °C, the cell migration inserts were removed, and the exterior was washed by dipping in a 24-well plate with PBS. We then used a PBS-wet cotton swab to remove non migrated cells from the inside surface of the insert; the cells that migrated across the porous membrane to the outer surface of the insert were fixed in ice-cold methanol for 10 min. The fixed inserts’ exteriors were then washed with PBS as above and incubated for 10 min in a 24-well plate with crystal violet (HT90132, Sigma-Aldrich, St. Louis, MO, USA) for staining. After PBS washing, inserts were air-dried and then placed into a 24-well plate containing 33% acetic acid for stain extraction. The optical density of equal amounts of the extracted stain solution was loaded in 96-well plates and read at 590 nm. The experiment was performed in duplicate for each condition and repeated four times. Additionally, some crystal violet-stained insert membranes were cut out using a scalpel and mounted on a glass slide in Vectamount Permanent Mounting Medium (H-5000, Vector Laboratories, Newark, CA, USA). Images were acquired using brightfield at 10× magnification.

### 4.5. Statistical Analysis

Each in vitro experiment was performed with a minimum of three technical repeats, and independently repeated at least three times, each with a corresponding calculated mean. Similarly, each animal experiment was performed with two technical repeats and independently repeated four times, each with a corresponding calculated mean. Results are presented as a final mean calculated from the corresponding mean results for each independent experiment ± standard error of means (SEM). GraphPad Prism 10 statistical software was used to calculate the statistical significance of these results. A paired Student’s *t*-test was used to determine statistical significance of results observed from experiments obtained from the same cell line while an unpaired *t*-test was used to determine statistical significance of results observed between distinct cell lines. A result with a *p*-value of less than 0.05 was considered statistically significant.

## Data Availability

The original contributions presented in this study are included in the article. Further inquiries can be directed to the corresponding author.
